# Considering the Role of Murine Double Minute 2 in the Cardiovascular System?

**DOI:** 10.3389/fcell.2019.00320

**Published:** 2019-12-10

**Authors:** Brian Lam, Emilie Roudier

**Affiliations:** Angiogenesis Research Group, School of Kinesiology and Health Sciences, Muscle Health Research Center, Faculty of Health, York University, Toronto, ON, Canada

**Keywords:** murine double minute 2 (MDM2), cardiovascular health, intracellular signaling, cardiac, endothelial, vascular smooth muscle cells

## Abstract

The E3 ubiquitin ligase Murine double minute 2 (MDM2) is the main negative regulator of the tumor protein p53 (TP53). Extensive studies over more than two decades have confirmed MDM2 oncogenic role through mechanisms both TP53-dependent and TP53-independent oncogenic function. These studies have contributed to designate MDM2 as a therapeutic target of choice for cancer treatment and the number of patents for MDM2 antagonists has increased immensely over the last years. However, the question of the physiological functions of MDM2 has not been fully resolved yet, particularly when expressed and regulated physiologically in healthy tissue. Cardiovascular complications are almost an inescapable side-effect of anti-cancer therapies. While several MDM2 antagonists are entering phase I, II and even III of clinical trials, this review proposes to bring awareness on the physiological role of MDM2 in the cardiovascular system.

## Introduction

Murine double minute 2 was identified in 1993 as a gene that is amplified in about one-third of human soft tissue sarcomas (Leach et al., 1993). This E3 ubiquitin ligase is one of the main negative regulators of the transcription factor tumor protein p53 (TP53). MDM2 regulates TP53 activity through direct interaction and ubiquitination, repressing its transcriptional activity (Levine and Oren, 2009; Vousden and Prives, 2009; Bieging et al., 2014). MDM2-TP53 interaction is highly regulated by multiple PTMs of both proteins (Meek and Anderson, 2009; Meek and Hupp, 2010), and through interactions with other binding partners (Naski et al., 2009; Fåhraeus and Olivares-Illana, 2014). MDM2 also has TP53-independent oncogenic properties (Bohlman and Manfredi, 2014, 2016). Thus, using antagonists that would limit MDM2 action represents a valuable anti-cancer approach, independently of the TP53 status of cancer cells (Toledo and Wahl, 2006; Bohlman and Manfredi, 2014, 2016). Most patents for MDM2 antagonists concern small molecule inhibitors, e.g., Nutlin-3a and RG7112. Some of these molecules are currently under phases I, II and III of clinical trials (Li and Lozano, 2013; Bohlman and Manfredi, 2016; Burgess et al., 2016). Since cardiovascular side effects of anti-cancer therapies are a recurrent problem (Jain et al., 2017), characterizing the cardiovascular function of MDM2 has become a pressing issue. Here, we propose to review the current literature regarding the cardiovascular function of MDM2. This review is divided into three parts. The first part analyzes whether the network of MDM2-interacting proteins plays a role in the cardiovascular system. The second part summarizes the knowledge gained from MDM2-transgenic mice. The last part examines the role of MDM2 in endothelial cells, in vascular smooth muscle cells (VSMCs) and in cardiomyocytes, some of the most important cells of the cardiovascular system.

## The Network of MDM2-Interacting Proteins and the Cardiovascular System

MDM2 is ubiquitous and regulates key cellular processes, such as differentiation, DNA repair and synthesis, transcription, intracellular trafficking, cell cycle, hypoxia signaling, apoptosis and oxidative stress response. To achieve these functions, MDM2 acts on TP53 and a myriad of other downstream proteins (Fåhraeus and Olivares-Illana, 2014). The activity of MDM2 is tightly regulated through PTMs that include ubiquitination, SUMOylation, putative acetylation and phosphorylation (Kubbutat et al., 1997; Iwakuma and Lozano, 2003; Meek and Anderson, 2009; Marine and Lozano, 2009; Naski et al., 2009; Meek and Hupp, 2010; Nag et al., 2013; Fåhraeus and Olivares-Illana, 2014). As a proof of concept that the MDM2 network plays a role in the cardiovascular system, we discuss here some evidence supporting the idea that upstream regulators (see Table 1 for summary) and downstream targets (see Table 2 for summary) of MDM2 can regulate the homeostasis of vascular beds and the cardiac tissue.

**TABLE 1:** Summary of the putative cardiovascular functions of upstream regulatory kinases for MDM2 discussed in this review.

**Kinase**	**Sites phosphorylated**	**Effect on MDM2 function**	**Known or presumed cardiovascular function**
DNA-PK	Ser17	Limits MDM2-TP53 interaction	Control of smooth muscle proliferation (Medunjanin et al., 2015; Kinoshita et al., 2017)
			Maintenance of endothelial cell quiescence (Mannell et al., 2010)
			Increased level of myocardial expression in dilated cardiomyopathy (Bartunek et al., 2002)
ERK1/2	Ser166 and Ser 186	Stabilizes MDM2, facilitates nuclear translocation	Alterations of the ERK related pathway are involved in cardiovascular pathogenesis (Muslin, 2008)
Akt/PKB	Ser166 and Ser 186	Stabilizes MDM2, limits self-ubiquitination and degradation, facilitates nuclear localization	Akt/PKB pathway is crucial regulator of cell survival, angiogenesis, vasodilation, metabolism in the cardiovascular system (Abeyrathna and Su, 2015)
Cyclin A/CDK2	Thr216	Promotes MDM2-TP53 interaction	Control of cell cycle in the cardiovascular system (Stanley-Hasnain et al., 2017)
GSK-3	Ser240 and Ser254	Limits MDM2-TP53 interaction, inhibition of TP53 ubiquitination and degradation	Regulates cardiac myocyte metabolism and controls cardiac hypertrophy (Potz et al., 2016; Takahashi-Yanaga, 2018)
ATM	Ser395	Reduces the capacity of MDM2 to facilitate the nuclear-cytoplasmic translocation and degradation of TP53	Control of pathological angiogenesis, involvement in atherosclerosis, insulin resistance and cardiac remodeling and sensing of β-adrenergic signals (Okuno et al., 2012; Espach et al., 2015)

**TABLE 2:** Summary of the putative cardiovascular functions of downstream effectors of MDM2 discussed in this review.

**Downstream effector**	**Effect on downstream targets**	**Known or presumed role of target in the cardiovascular system**
ARC, Apoptosis Repressor with Caspases recruitment domain	MDM2 promotes the degradation of ARC	ARC has anti-apoptotic function in cardiomyocytes and skeletal muscles (Koseki et al., 1998)
β-arrestin	MDM2 binds, interacts and ubiquitinates with β-arrestin 1 proteins.	Binding between β-arrestin1 and MDM2 restrains p53 activity (Hara et al., 2011, 2013)
	MDM2 ubiquitination facilitates binding to signaling kinases downstream of β-arrestin 2	Ubiquitination of β-arrestin is crucial to regulate internalization and recycling of β-adrenergic receptors (Shenoy et al., 2007; Jean-Charles et al., 2016)
E2F1, E2F transcription factor 1	MDM2 binding stimulates the activity of E2F1	E2F1 promotes cardiac dysfunction and increases the size of infarction after myocardial infarction (MI).
		E2F1 stabilizes TP53 and represses the expression of VEGF-R2, facilitating apoptosis in endothelial cells and inhibition of angiogenesis after MI (Wu et al., 2014)
FoxO1, Forkhead box protein O1	MDM2 binds and interacts with FoxO1, triggering its degradation by the proteasome	Endothelial FoxO1 promotes an angio-static environment in ischemic tissue (Milkiewicz et al., 2011; Roudier et al., 2013)
		FoxO1 expression supports diabetic cardiomyopathy (Battiprolu et al., 2012; Qi et al., 2015; Chistiakov et al., 2017)
		FoxO1 limits endothelial cell migration (Aiken et al., 2016)
FoxO4, Forkhead box protein O4	MDM2 favors both mono- and poly-ubiquination of FoxO4. Mono-ubiquitination stabilizes FoxO4. Poly-ubiquitination triggers FoxO4 degradation.	Endothelial FoxO4 promotes inflammation and cardiac dysfunction after myocardial infarction (Zhu et al., 2015)
GRK2, G-protein-coupled receptor kinase 2 (i.e., β-adrenergic receptor kinases 1)	MDM2 ubiquitinates GRK2 triggering its proteasomal degradation.	Absence of MDM2 regulation of GRK2 induces GRK2 stabilization, uncoupling of β-adrenergic receptor and G protein, the β-adrenergic receptor stays in a desensitized state (Jean-Charles et al., 2017)
HDAC1, histones deacetylase 1	MDM2 ubiquitinates HDCA1	Reduction of HDAC1 protein level by MDM2 supports vascular calcification *in vivo* and *in vitro* in vascular smooth muscle cells (Kwon et al., 2016)
HIF1-α, hypoxia-inducible factor 1 α	MDM2 stabilizes HIF1α in an Akt-dependent manner	Lack of HIF1-α during cardiac hypertrophy supports transition to heart failure due to a lack of angiogenesis (Sano et al., 2007; Toko et al., 2010)
NICD, Notch intracellular domain	MDM2 interacts with and ubiquitinates NICD. MDM2 ubiquitination of NICD is an activation signal rather than a trigger for degradation by the proteasome (Pettersson et al., 2013).	Inhibition of endothelial NOTCH causes heart hypertrophy and heart failure due to impaired transport of fatty acids and altered blood vessel growth (Jabs et al., 2018)
TP53, tumor protein p53	MDM2 binding to p53 limits TP53 transcriptional activity.	TP53 provokes transition from cardiac hypertrophy to heart failure by promoting cardiomyocyte apoptosis and limiting angiogenesis (Sano et al., 2007; Toko et al., 2010)
	MDM2 ubiquitination triggers proteasomal degradation of TP53	Preserving MDM2 action on p53 prevents ischemic limb loss after femoral artery ligation (Morimoto et al., 2011)
		TP53 limits vascular smooth muscle cells proliferation in a pro-atherogenic environment (Hashimoto et al., 2011)
Tip60, Histone acetyltransferase KAT5	MDM2 interacts with and ubiquitinates Tip60 triggering Tip60 degradation	Tip60 regulates senescence, restrains cell cycle and is required for survival in cardiomyocytes in response to pressure overload (Fisher et al., 2012; Fisher et al., 2016; Hu et al., 2009)

### MDM2-Upstream Regulators

**FIGURE 1:**
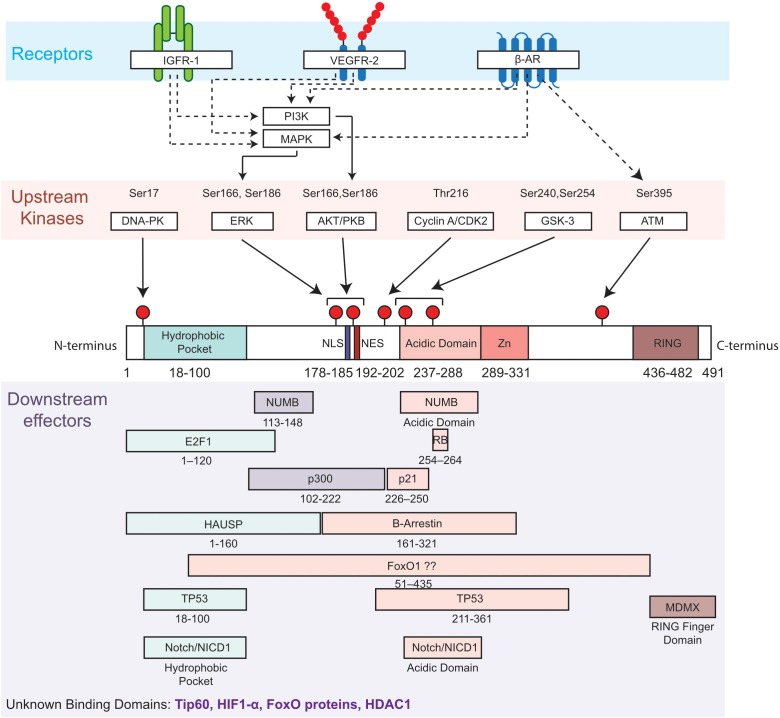
Diagram of MDM2 domains showing phosphorylation sites and upstream regulators (kinases and related receptors) of MDM2 functions, as well as putative binding sites with downstream effectors. Top, most common phosphorylation sites on Serine (Ser) and Threonine (Thr) are indicated by a red pin. Residues are numbered based on amino acid sequence from N-terminus to C-terminus. Middle, the functional domains of MDM2 include the hydrophobic pocket, the nuclear localization sequence (NLS), the nuclear export sequence (NES), the acidic and central domain, the Zinc domain (Zn) and the ring domain (RING). Bottom, selected interactions between MDM2 and its downstream effectors of MDM2 are illustrated. β-Adrenergic receptor, β-AR; DNA-dependent protein kinase, DNA-PK; E2F transcription factor 1, E2F1; extracellular-signal-regulated kinase, ERK; Forkhead box protein, FoxO; herpes virus-associated ubiquitin specific protease, HAUSP; Insulin-like growth factor receptor 1, IGFR1; Mitogen-activated protein kinases, MAPK; Mouse double Minute 4, MDM4; NUMB; Notch intracellular domain, NICD; Phosphotidylinositol 3-kinase, PI3K; Protein kinase B, PKB; protein numb homolog, Retinoblastoma protein, RB; Vascular Endothelial Growth Factor Receptor 2, VEGFR2.

Several upstream regulators of MDM2 are important actors of the cardiovascular homeostasis. To illustrate the idea, we have deliberately chosen to discuss the role of a few receptors and kinases (Figure 1).

We will first present how ligands relay their signals to MDM2 by discussing the role of two tyrosine kinases receptors, the Vascular Endothelial Growth Factor Receptor (VEGFR2) and the Insulin-like Growth Factor 1 Receptor (IGFR1), as well as one G-protein coupled receptor, the β-Adrenergic Receptor (β-AR).

Kinases sensing DNA damage inhibit MDM2, while growth factors activate MDM2 through the modulation of phosphorylation on multiple sites: the nuclear localization signal, the nuclear export signal, the acidic and central domain (Figure 1). For example, growth factors stimulate MDM2 functions by limiting glycogen synthase kinase-3 (GSK3)-dependent phosphorylation on the acidic domain (Serines 240 and 254) and by enhancing Akt-dependent phosphorylation (Serines 166 and 186). These actions of growth factors counterbalance the effects of DNA damage kinases, such as ATM which phosphorylates MDM2 on Serine 395 (Meek and Anderson, 2009; Meek and Hupp, 2010). Therefore, we will analyze the specific role of the ataxia telangiectasia mutated (ATM) kinase, a DNA damage kinase, and the role of GSK3 and Akt in the cardiovascular system.

#### Receptors Upstream of MDM2

The Vascular Endothelial Growth Factor-A (VEGF-A) is a very potent stimulator of angiogenesis, crucial to maintaining vascular homeostasis. VEGF-A binds to VEGFR2, a tyrosine kinase receptor, leading to its activation by auto-phosphorylation. Once activated, VEGFR2 relays its signal to downstream kinases including the Phosphotidylinositol 3-Kinase/Akt (PI3K/Akt) and the Mitogen-activated protein kinases (MAPKs) (Claesson-Welsh, 2016). Both the PI3K/Akt and MAPK pathways can phosphorylate MDM2 on serine residues 166 and 186 (Mayo and Donner, 2001; Feng et al., 2004; Hogan et al., 2008) to promote the oncogenic function of MDM2 in cancer cells. In a physiological context, we reported that VEGF-A promotes MDM2 phosphorylation. Indeed, VEGF-A elicits MDM2 phosphorylation in the skeletal muscle during physical exercise, as well as in primary endothelial cells (Aiken et al., 2016) (for more detailed information see Part 3 ‘Role of the MDM2 pathway in primary endothelial cells’).

IGFR1 and β-AR are important for cellular processes in the heart such as hypertrophy, contractility and cell survival (Stiles et al., 1984; Weeks et al., 2017). Binding of IGF1 to its receptor, IGFR1, promotes the phosphorylation of MDM2 through the Akt and MAPK pathways. This leads to MDM2 nuclear localization and enhances its stability and activity (Ogawara et al., 2002; Feng et al., 2004; Worrall et al., 2017). B -adrenergic stimulation is well known to activate both Akt and MAPK pathways (Morisco et al., 2000; Vidal et al., 2012). Most recently, chronic exposure to β-adrenergic agonist, isoproterenol, was reported to increase the expression of ATM, a kinase upstream of MDM2 (Foster et al., 2011). This suggests that MDM2 can sense the β-adrenergic and IGF1 signals. Interestingly, several publications highlight a more complex relationship. Indeed, MDM2 can regulate the trafficking of both IGFR1 and β-AR, modulating the sensitivity of cells to the IGF1 and catecholamines, respectively (Girnita et al., 2007; Jean-Charles et al., 2017). The relationship between MDM2 and β-AR is discussed in further detail in Part 3, ‘MDM2 and the regulation of the β-adrenergic signal.’

The few studies discussed above support the idea that MDM2 can relay the signals sensed by tyrosine kinase receptors and G-protein coupled receptors within the cardiovascular system. Yet, further investigations are warranted to fully elucidate how these receptors regulate MDM2 functions.

#### Cardiovascular Function of Ataxia Telangiectasia Mutated (ATM) Kinase

Patients with Ataxia telangiectasia syndrome not only present growth retardation, premature aging and a high risk of cancer, they also exhibit insulin resistance, glucose intolerance and a higher risk of ischemic heart disease (Espach et al., 2015). This suggests that ATM has cardiovascular functions. Interestingly, ATM has emerged as a regulator of atherosclerosis and potentially angiogenesis.

In the atherogenic environment of the Apo null background, haplodeficient *ATM*^±^ mice present the earliest onset of atherosclerosis (Mercer et al., 2012). Haplodeficient *ATM*^±^ mice also have worse cardiac remodeling and an impaired ventricular function post-myocardial infarction compared to their wild-type littermate, suggesting that ATM depletion facilitates the development of heart failure (Daniel et al., 2014, 2016; Jia et al., 2017). This suggests a potential role for ATM in maintaining cardiovascular health through the protection against atherosclerosis and pathological cardiac remodeling. Beyond these protective effects, ATM might regulate angiogenesis. It is well known that in response to DNA damage, ATM phosphorylates MDM2 on the Ser395 (de Toledo et al., 2000; Gannon et al., 2012). After hypoxia-reoxygenation, reactive oxygen species (ROS) activate ATM in immature blood vessels. The activation of ATM represses the p38α MAPK. Usually, p38α is activated by the VEGFR2 to support endothelial cell migration; but when overactivated p38α can favor endothelial cell apoptosis. ATM activity can limit p38α proapoptotic functions, thus enhancing endothelial survival (Okuno et al., 2012). Through this mechanism, ATM supports pathological angiogenesis in ischemic retinopathy and tumor growth. Since ATM knock-out in the whole body and specifically in the endothelium protects mice from these pathological forms of angiogenesis, it appears that ATM is a key regulator of angiogenesis driven by hypoxia and oxidative stress. We have reported a central role for MDM2 in muscle angiogenesis during endurance exercise (Roudier et al., 2012; Aiken et al., 2016), a stress-associated oxidative stress and potentially tissue hypoxia. Yet, it remains unknown whether the angiogenic function of MDM2 is regulated by ATM.

A lot remains to be discovered regarding the cardiovascular functions of ATM, however, this DNA damage kinase might modulate how the cardiovascular system adapts to micro-environmental stressors, such as oxidative stress, proatherogenic factors and ischemia.

#### Growth Factor Responsive Kinases

Growth factors stimulate MDM2 in multiple ways, including the downregulation of GSK3 and the activation of Akt. Interestingly, both GSK3 and Akt play an important role in controlling cardiac hypertrophy (Weeks et al., 2017; Takahashi-Yanaga, 2018).

GSK3 is ubiquitous and normally active in the absence of growth factors. GSK3 phosphorylates the central domain of MDM2, decreasing MDM2 actions on its targets, e.g., TP53 (Kulikov et al., 2005; Lin et al., 2012). To support cardiac function during hypertrophy, it is crucial that angiogenesis occurs to maintain proper blood supply to the cardiac muscle. Chemical inhibition of GSK3 by IM-12 improves myocardial blood flow recovery in pigs fed with a high-fat diet then subjected to myocardial infarction induced by left circumflex coronary artery constriction (Potz et al., 2016). Overall, the increased capillary density suggests improved tissue perfusion. Unfortunately, the capillary-to-cardiomyocytes ratio was not reported in this study. The observed increase in myocardial capillary density could be a result of reduced hypertrophy, since GSK3 is a negative regulator of cardiac hypertrophy (Takahashi-Yanaga, 2018). Indeed, reduced cardiomyocyte size would increase capillary density without any gain of capillaries. Therefore, reporting a capillary-to-cardiomyocytes ratio is crucial to demonstrate whether angiogenesis occurred.

Growth factors induce Akt-dependent phosphorylation of MDM2. This promotes MDM2 interaction with the HIF1-α, stabilizing HIF1-α and enhancing its transcriptional activity on pro-angiogenic genes (Mayo and Donner, 2001; Bárdos et al., 2004). Akt-dependent phosphorylation of MDM2 facilitates MDM2 interaction with TP53, leading to TP53 ubiquitination and degradation. The resulting decrease in TP53 restrains apoptosis and can favor angiogenesis by limiting angiostatic gene expression, such as Thrombospondin-1 (Dameron et al., 1994). It is worth noting that TP53 and HIF1-α can compete for binding to MDM2, which means that under hypoxia HIF1-α could restrain the capacity of MDM2 to inhibit TP53 (Robertson et al., 2014).

In the heart, Akt signaling coordinates the growth of cardiomyocytes with angiogenesis to ensure the cardiac tissue remains well perfused during physiological hypertrophy (Weeks et al., 2017). Conversely, pathological hypertrophy promotes TP53 expression. Cardiac hypertrophy induced by pressure overload leads to TP53 accumulation, restriction of HIF1-α functions and a lack of angiogenesis (Sano et al., 2007); a similar outcome was observed in the failing heart (Toko et al., 2010). While it remains unknown whether Akt-dependent phosphorylation of MDM2 plays a role in these pathological changes in TP53 and HIF1-α, a study suggests that increased MDM2 activity could be beneficial. Indeed, overexpression of IGF1 *in vitro* and *in vivo* enhanced MDM2 activity toward TP53 in cardiomyocytes preventing apoptosis and the activation of the renin-angiotensin system, a hallmark of heart failure (Leri et al., 1999a, b).

In the KK-Ay diabetic mice, atorvastatin, a cholesterol-lowering drug, preserved the insulin sensitivity of Akt in the lower limb after femoral artery ligation. Atorvastatin increased the levels of Serine 166 phosphorylation and prevented TP53 accumulation in the ischemic lower limb. Since atorvastatin rescued limb loss after femoral artery ligation. These data suggest that activation of MDM2 supports limb recovery (Morimoto et al., 2011).

GSK3 seems to support adverse remodeling after myocardial infarction, while Akt facilitates beneficial adaptations. Additional investigations are required to fully comprehend how GSK3 and Akt impact MDM2 function in the context of these cardiovascular diseases. Together these studies, however, clearly support that kinases upstream of MDM2 play a role in controlling the balance between microvascular content and striated muscle hypertrophy during pathological remodeling.

### MDM2-Downstream Targets

MDM2’s functions go beyond the old paradigm that MDM2 is a key regulator of transcriptional factor TP53. Indeed, this E3 ubiquitin ligase has numerous targets in all cellular compartments. Therefore, we have chosen to discuss several examples of MDM2 targets, including some nuclear proteins involved in the regulation of gene expression and several cytoplasmic proteins involved in key intracellular signaling and trafficking pathways.

#### Nuclear Downstream Targets of MDM2

##### MDM2-FoxO proteins interaction

We and others have reported that MDM2 is key regulator of FoxO transcription factors (Horst et al., 2006; Brenkman et al., 2008; Yang J.-Y. et al., 2008; Yang W. et al., 2008; Fu et al., 2009; Aiken et al., 2016). The original observation that FoxOs are important for longevity (Salih and Brunet, 2008), led researchers to explore the cardiovascular functions of FoxO. FoxO proteins are well-known regulators of antioxidant defense. With no exception, FoxO1 and FoxO3 have been reported to be important to maintain antioxidant molecules (i.e., catalase and SOD2) in response to cardiac ischemia-reperfusion (Sengupta et al., 2011; Puthanveetil et al., 2013). Due to this function, FoxO proteins were thought to have protective properties in the cardiovascular system. However, this might be context- and tissue-dependent.

In the vasculature, endothelial knock-down of FoxO1, FoxO3 and FoxO4 appears to be protective against atherosclerosis in LDLr^–/–^ mice, a transgenic mouse model that creates a pro-atherogenic environment due to a lack of low-density lipoprotein receptors (LDLr) (Tsuchiya et al., 2012). This supports the detrimental role of FoxO protein in arteries exposed to an atherogenic environment. Mouse models of type 2 diabetes present increased levels of cardiac FoxO1 (Battiprolu et al., 2012; Qi et al., 2015; Chistiakov et al., 2017). This increase in FoxO1 downregulates insulin receptor substrate 1 (IRS-1) impairing Akt activity and insulin sensitivity. Cardiac-specific knock-down of FoxO1 prevented the development of diabetic cardiomyopathy, making a strong case for a detrimental role of cardiac FoxO1 during diabetic cardiomyopathy. FoxO1, β-arrestin, IRS and IGF1R are all crucial to maintaining an efficient insulin signal (Usui et al., 2004; Girnita et al., 2007). Interestingly, these proteins might compete for binding to MDM2 (Balaji et al., 2018). Yet, the role of cardiac MDM2 in diabetic cardiomyopathy remains unclear. In the context of post-myocardial infarction, endothelial-specific depletion of FoxO4 improved cardiac function by preventing loss of nitric oxide production. Maintenance of nitric oxide restrained inflammation by suppressing monocyte adhesion (Zhu et al., 2015). Interestingly, MDM2 is upregulated in the cardiac tissue 24 h post-myocardial infarction and remains elevated up to 4 weeks post-infarction (Zhao et al., 2016). MDM2 inhibition by Nutlin-3a improved survival and repressed the inflammatory response in this context. MDM2 has been reported to prime the mono-ubiquitination of FoxO4 in response to oxidative stress (Brenkman et al., 2008) leading to FoxO4 poly-ubiquitination and degradation when MDM2 levels are high (Hauck et al., 2017). It is then appealing to hypothesize that the interaction between MDM2 and FoxO4 in the endothelium controls the inflammatory response after myocardial infarction; a hypothesis that remains unverified. Finally, we reported an upregulation of FoxO1 in ischemic skeletal muscle during peripheral artery disease (Milkiewicz et al., 2011; Roudier et al., 2013) that correlated with a restrained binding between MDM2 and FoxO1. Endothelial-directed depletion of FoxO1 enhanced angiogenesis (Milkiewicz et al., 2011). Since constitutive active MDM2 reduces FoxO1 transcriptional activity in endothelial cells (Aiken et al., 2016), it is appealing to hypothesize that MDM2 impedes the angiostatic function of FoxO1 in the endothelium.

Based on the above studies it appears that an in-depth analysis of the FoxO-MDM2 interaction in the cardiovascular tissues has the potential to help fully comprehend how the FoxO functions (i.e., antioxidant defense, angiostasis, inflammation, etc.) are regulated during cardiovascular diseases.

##### MDM2, E2F1 and the ischemic heart

E2 promoter binding Factor 1 (E2F1) is a transcriptional activator mainly known to regulate the cell cycle, apoptosis and angiogenesis to support tumor progression (Schaal et al., 2014). MDM2 was shown to stimulate E2F1 transcriptional activity in tumors (Martin et al., 2014), potentially shifting E2F1 properties from antiproliferative to proliferative (Loughran and Thangue, 2000). Interestingly, E2F1-deficient mice show improved cardiac function, reduced size of myocardial infarction, and enhanced post-infarction cardiac angiogenesis after ligation of the left anterior descending coronary artery. These beneficial effects of E2F1 deficiency in the ischemic heart were due to an upregulation of pro-angiogenic factors (i.e., vascular endothelial growth factor A, VEGF-A and placental growth factor, PIGF (Wu et al., 2014). In cardiac fibroblasts subjected to hypoxia, the overexpression of E2F1 increased the expression of TP53, which was required to repress the expression VEGF-A; while a decrease in PIGF occurred independently of the TP53 status of these cells. These results suggested that E2F1 promotes the expression of pro-angiogenic factors through both TP53-dependent and TP53 independent mechanisms (Wu et al., 2014). Conversely to previous observations made in tumor cells, MDM2-deficiency in cardiac cells was reported to enhance E2F1 transcriptional activity and to have detrimental effects on the heart homeostasis (Hauck et al., 2017) (see below Part ‘Insight from MDM2 transgenic mice’). Further investigations are thus warranted to better understand how the interplay between MDM2 and E2F1 impacts myocardial physiology and recovery post-infarction.

##### MDM2, Tip60, and cardiac hypertrophy

Tip60 is an acetyltransferase that acetylates histones and other proteins, including TP53 and ATM. When TP53 is acetylated on lysine 120 by Tip60, it triggers TP53-dependent apoptosis (Tang et al., 2006). MDM2 can interact with Tip60. MDM2 promotes the proteasomal degradation of Tip60 (Legube et al., 2002), whereas Tip60 binding to MDM2 restrains the neddylation capacity of the E3 ligase (Dohmesen et al., 2008). Tip60 expression in cardiac tissue is enriched during early cardiac development and adult hearts are reported to express Tip60 (Lough, 2002; Hu et al., 2009; Fisher et al., 2012, 2016). Tip60^±^ heterozygous mice (12–14 week-old) present an enhanced cell cycle activity and reduced apoptosis, that could support better cardiac regeneration in the heart subjected to pressure-overload (Fisher et al., 2012).

Specific depletion of Tip60 in cardiomyocytes in mice increased cardiomyocyte cell cycling *in vivo* in 2–4 week-old hearts (Fisher et al., 2016). However, this was followed by a significant loss of cardiomyocytes after 8 weeks, a reduction of heart mass leading to death between 8 and 12 weeks of age due to cardiac dysfunction. Together these results suggest that Tip60 is crucial to control the balance between cell cycle and apoptosis in the myocardial cells. Yet, the role of MDM2 on the cardiac functions of Tip60 remains to be studied.

#### Cytoplasmic Downstream Targets of MDM2

##### MDM2, endothelial Notch and cardiac hypertrophy

The NOTCH pathway acts conjunctly with Vascular Endothelial Growth Factor-A (VEGF-A) to allow patterning of blood vessels during sprouting angiogenesis (Blanco and Gerhardt, 2013). This is essential for vascular growth. An elegant study by Jabs et al. (2018) recently demonstrated the role of the NOTCH pathway in the adult endothelium of the heart. Endothelial-specific downregulation of NOTCH1 signal was induced by conditional ablation of the Rbp-jκ complex in adult mice. In this model, restriction of the endothelial NOTCH pathway was highly associated with cardiac dysfunction, cardiac hypertrophy and impairment of fatty acid transport through the endothelium. The source of energy shifted in favor of glucose instead of fatty acids, likely limiting the capacity to supply ATP for cardiac function. All these changes are hallmarks of heart failure. Down-regulation of the NOTCH pathway was associated with an increase of cardiac microvascular density, supporting the idea that NOTCH imposes a brake on cardiac angiogenesis (Jabs et al., 2018). Usually improved cardiac angiogenesis is associated with physiological cardiac hypertrophy. The observation that enhanced angiogenesis led to heart failure supports the idea that the growth of an abnormal vascular bed occurs when NOTCH is inhibited. The main negative regulator NOTCH1 is NUMB. Indeed, NUMB facilitates NOTCH1 ubiquitination and degradation. MDM2 positively regulates the NOTCH signaling pathways through its capacity to bind to both NUMB and to the NICD1. MDM2 promotes the degradation of NUMB, an event that could enhance NOTCH1 signaling. Conversely, MDM2 binds to the NICD1. This binding does not promote the ubiquitination nor the degradation of NOTCH1, but rather it activates the NOTCH signaling pathway, in cancer cell lines (Pettersson et al., 2013). Since we and others have reported pro-angiogenic functions of MDM2 (Secchiero et al., 2007; Roudier et al., 2012), further investigations are required to decrypt the mechanisms controlling endothelial NOTCH activity in the adult heart, studying the putative involvement of MDM2 might bring new insight on how a normal cardiac microvasculature is established.

##### MDM2 and beta-adrenergic pathway

MDM2 is associated with key regulators of the beta-adrenergic pathway through its capacity to interact with β-arrestin, a protein adapter of G protein-coupled receptor and G Protein-Coupled Receptor Kinase 2 (GRK2) (Jean-Charles et al., 2017). The pivotal work done by Hara et al. (2011, 2013) clearly established that chronic exposure to β-adrenergic catecholamines facilitates MDM2-β-arrestin1 interaction, restraining TP53 activity. One consequence could be an impairment of the DNA damage response with a potential impact on genomic integrity (Hara et al., 2013). Additional works also support the idea that MDM2 might function to recycle G protein-coupled receptors; playing a key role in the desensitization and re-sensitization of these receptors, including the β-AR (Wang et al., 2003; Shenoy et al., 2009; Jean-Charles et al., 2016, 2017). Ultimately, this is supportive of a role for MDM2 in the cardiovascular system through the modulation of the cardiac response to β-adrenergic stimulation (Cannavo et al., 2013).

### Summary of “MDM2’s Social Network” and the Cardiovascular System

The studies presented above, while not exhaustive, serve as examples to highlight the diverse role of MDM2 as a central hub in the cardiovascular system. Most human cardiovascular diseases are associated with DNA damage due to oxidative stress (Jackson and Bartek, 2009; Shimizu et al., 2014; Uryga et al., 2016) and alteration of hormonal signals (Higashi et al., 2019). The DNA damage kinase ATM is an upstream regulator of MDM2, and it does modulate the endothelial function, playing a role in atherosclerosis and angiogenesis. Growth factor responsive kinases known to phosphorylate MDM2 appears as crucial for a proper balance between vascularization and hypertrophy during cardiac remodeling. MDM2 cardiovascular functions are likely TP53- and HIF1-α-dependent, however, numerous TP53-independent MDM2 binding partners also emerge as key actors of cardiac and vascular homeostasis. Yet, a lot remains to be done to fully understand how the “MDM2 social network” participates in cardiovascular homeostasis over the lifespan (Fåhraeus and Olivares-Illana, 2014).

## Part 2: Insight From the MDM2 Transgenic Mice

The phenotypic analyses of MDM2 transgenic mouse models further demonstrate a crucial role for MDM2 in the cardiovascular system (Table 3).

**TABLE 3:** Description and summary of the cardiovascular phenotypes observed in MDM2 transgenic mouse model.

**Transgenic mice model**	**Type of genetic modification**	**Site of transgenic modification**	**Cardiac or vascular phenotype**
MDM2-null	MDM2 null mice. MDM2 mutant lacking exon 10–12 or 7–12 were.	Whole body	Embryonic lethality occurs before cardiac development (Jones et al., 1995; Montes de Oca Luna et al., 1995)
MDM2 transgenic mice	Full length MDM2 overexpression under the control of its native promoter region. fourfold increase in MDM2 mRNA expression. MDM2 transgenic mice were generated in both p53 wild-type and p53-null backgrounds	Whole body	Independent of p53 background, mice with MDM2 transgene have a higher proportion of hemangiosarcoma than p53-null mice with normal levels of MDM2 (Jones et al., 1998).
Hypomorphic MDM2 mice	Downregulation of protein expression by 70% using a hypomorphic allele MDM2^*puro*^ and a recombined null allele MDM2^Δ^ ^7–9^ (Mendrysa et al., 2003)	Whole body	Capillary rarefaction and lack of angiogenesis in response to physiological stimulus in the skeletal muscle (Roudier et al., 2012)
			Enhanced cardiac/cardiomyocyte hypertrophy, and increased alteration of myocardial tissue and function after ischemia (Toth et al., 2006)
Endothelial MDM2-KO mice	Tie2-Cre driven knock-down of MDM2 in endothelial cell lineage	Endothelial lineage cells	Embryonic lethality at E10.5 due to vascular remodeling defect, i.e., enlargement of the aortic lumen, the cardinal veins and the extraembryonic vasculature (Zhang et al., 2014).
Cardiomyocyte MDM2-KO mice	α*MyHC*-Cre transgene was used to induce MDM2 deletion in cardiomyocytes	Cardiomyocytes	Embryonic lethality at E9.5 failure to develop a functional heart (Grier et al., 2006)
Inducible Endothelial MDM2-KO mice i-EC-MDM2KO	Cross breeding of *Pdgfb*-Cre-ER mice with mice harboring the floxed MDM2 alleles	Induction of endothelial-specific MDM2 deletion by tamoxifen	Abnormal swelling of animals 5 days after the injection of tamoxifen Death of all i-EC-MDM2-KO mice 16 days after tamoxifen injection (Yokoyama et al., 2019)
Inducible Cardiomyocyte specific MDM2-KO mice	Cardiac muscle a-myosin heavy chain 6 promoter drives the expression of Cre under the control of Tamoxifen Tg(Myh6-Cre/Esr1)	Induction of cardiomyocytes deletion by hydroxytamoxifen.	Cardiomyocytes-specific deletion of MDM2 lead to spontaneous concentric hypertrophy and cardiac dysfunction and early mortality (Hauck et al., 2017).
Inducible smooth muscle specific MDM2-KO mice i-SMC-MDM2-KO mice	Cross breeding of SMC-specific *SM22* promoter (SM-CreER^T2^) mice with mice harboring the floxed MDM2 alleles	Induction of smooth muscle cell-specific MDM2 deletion by tamoxifen	Cre activity was not found in the cardiovascular system nor in the aorta. Death of all i-SMC-MDM2-KO mice 12 day post tamoxifen injection due to gastrointestinal alterations (Boesten et al., 2006).

### Vascular Phenotype and MDM2

Over the last two decades, several transgenic mouse models have been generated to study the function of MDM2, revealing a crucial role of MDM2 in supporting survival, as well as vascular function.

Full *Mdm2* deletion results in embryonic lethality at E5 (Jones et al., 1995; Montes de Oca Luna et al., 1995). Embryonic lethality was completely reversed in double-null mice, harboring both MDM2 and TP53 deficiency (*Mdm2*−/− and *Trp53*−/−). Due to the early stage of lethality, these initial studies did not allow for the examination of MDM2 function in adult cardiac tissue. To override the lethality observed in mice with full MDM2 knock-down, Zhang et al. (2012) created a transgenic mouse with a selective knock-down of MDM2 in the endothelial cell lineage using the Tie2-Cre transgene. The loss of endothelial MDM2 also resulted in embryonic lethality, however, at a later stage (E10.5). This lethality was due to defective vascular patterning, including an enlargement of the aorta, the cardinal veins, and the extraembryonic vasculature (Zhang et al., 2014). The expression of TP53 targets genes, *p21*, *Nuxa* and *Puma*, was increased at E8.5 and E9.0 in heart tube and apoptosis was enhanced. Most recently, to study the role of MDM2 in the adult endothelium, Yokoyama et al. (2019) generated a transgenic mouse model in which the inducible endothelial cell-specific depletion of MDM2 was achieved by tamoxifen injection using the *Pdgfb*-Cre-ER transgene in 8-week old mice. Five days after the injection of tamoxifen, some abnormal swelling was observed in mice with endothelial-specific deletion of *Mdm2*. All mice died 16 days post-injection. This phenotype was associated with severe increases in TP53 expression and apoptosis in the vascular bed, including in the skeletal muscle and the heart. MDM2 appeared as crucial to maintain the vascular barrier and permeability function of the endothelium. Taken together, these studies present a strong case that in the embryo and in adult mice, MDM2 acts in a TP53-dependent manner to limit excessive TP53 overactivation and apoptosis in tissues, including in the endothelium.

While the primary function of MDM2 might be to restrain TP53, additional evidence supports the idea that MDM2 is important for vascular homeostasis due to TP53-independent functions. MDM2 overexpression in mice elicits tumor formation, bringing irrefutable evidence that MDM2 promotes cancer development. A close phenotypic analysis of these mice also reveals an endothelial function of MDM2. The mice overexpressing MDM2 in a *Trp53* null background presented a unique subset of tumors compared to mice overexpressing MDM2 in a *Trp53* wild-type background. Under a *Trp53* null background, a higher rate of hemangiosarcoma was observed. This type of tumor is due to an uncontrolled proliferation of endothelial cells (Jones et al., 1998). These observations suggest that MDM2 can support endothelial cell proliferation independently of simply controlling TP53 activity.

Another very interesting transgenic rodent model is the hypomorphic *Mdm2*^puro/Δ^ ^7–9^ mouse. These mice harbor one hypomorphic allele (puro) and one null allele (Δ7-9), thus leading to a reduction of MDM2 expression by 70% of the normal level of expression in tissue (Mendrysa et al., 2003). Under basal conditions, these mice do not present TP53 overactivation. *Mdm2* hypomorphic mice, however, have a reduction in MDM2 activity. We reported a decrease of the capillary to fiber ratio by 20% in the skeletal muscle of the hypomorphic *Mdm2*^puro/Δ^ ^7–9^ mice (Ogawara et al., 2002) independently of TP53 status (Ogawara et al., 2002). In response to endurance training, a well-established powerful physiological stimulus for muscle angiogenesis, the development of new blood vessels was blunted in exercising *Mdm2*^puro/Δ^ ^7–9^ mice. This shows that normal expression of MDM2 is required both for the maintenance and the expansion of the vascular bed. These studies support the notion that beyond its primary function to keep TP53 under control, MDM2 has TP53-independent vascular functions that impact the capacity of endothelial cells to proliferate and to initiate angiogenesis.

The smooth muscle tissue is another key component of blood vessels. Mice with smooth muscle-specific MDM2 deficiency have been generated using a tamoxifen-inducible Cre under the control of the *SM22* promoter (Boesten et al., 2006). These mice died shortly (9–13 days) after the induction of MDM2 depletion in the smooth muscle. However, the depletion of MDM2 in the aorta was not be achieved with this approach, the author did not investigate potential depletion in other vascular structures (i.e., arterioles). So, it remains impossible to conclude regarding the *in vivo* role of MDM2 in the vascular smooth muscle. The putative role of MDM2 in cultivated VSMCs is discussed in Part 3 of this review.

### Cardiac Phenotype and MDM2

MDM2 appears to be crucial for the embryonic development of the heart. Tissue-specific loss of MDM2 in cardiomyocytes induced by the α*MyHC*-Cre transgene results in embryonic lethality. At E9.5, embryos with cardiomyocyte-specific *Mdm2* deletion failed to develop a functional heart (Grier et al., 2006). Immunohistochemistry staining of cardiomyocytes revealed a significant thinning of the myocardial layer of the ventricles and overall a much smaller heart weight, probably due to an increased level of apoptosis. By E13.5, no embryos survived, and most of them lacked visible signs of heart development (Grier et al., 2006).

Interestingly, hypomorphic *Mdm2*^*puro*^/^Δ^^7–9^ mice present a lower heart weight, supporting the notion that MDM2 is required to maintain heart mass. However, the heart-to-body mass ratio, as well as the cardiomyocyte cross-sectional area were increased in the hypomorphic mice (Toth et al., 2006). These mice were also more sensitive to ischemia-induced damage presenting an increased infarction area and an impaired cardiac function (Toth et al., 2006). To study specifically the function of MDM2 in the adult heart, Hauck et al. (2017) generated mice with a cardiomyocyte-specific inducible depletion of *Mdm2*. This ablation of *Mdm2* in the cardiomyocytes led to left ventricular dysfunction and concentric remodeling of the heart, as shown by a 98% increase in the heart-to-body mass and a 1.9-fold increase in cardiomyocyte size 18 days after 4-hydroxytamoxifen injection (Hauck et al., 2017). Mitochondrial dysfunctions were also observed due to a lack of proper mitochondria biogenesis. These mitochondrial effects were a consequence of alterations in the transcription of TP53-dependent genes involved in mitochondria energy metabolism (*Pgc1*-α, *Ppar*-α/γ and *Esrr*-β/γ) (Hauck et al., 2017). Interestingly, *Mdm2* mRNA levels were found to be reduced in patients with ischemic cardiomyopathy, idiopathic dilated cardiomyopathy and chemotherapy-induced cardiomyopathy (Toth et al., 2006). Together these data support the idea that reduced expression of MDM2 may facilitate cardiomyocyte hypertrophy and cardiac hypertrophy.

### Summary of Knowledge Gained From MDM2-Transgenic Mouse Models

In the embryo, MDM2 is essential to both the cardiac and endothelial tissue development with a primary role as a negative regulator of TP53-induced apoptosis. In adult mice, MDM2 appears essential for controlling cardiac hypertrophy, potentially through TP53 dependent mechanisms (Toth et al., 2006; Hauck et al., 2017). Loss of MDM2 in the endothelium is lethal in adult mice due to TP53 overactivation. However, MDM2 appears to have TP53-independent functions that regulate vascular homeostasis. Too little expression of MDM2 supports vascular rarefaction and lack of angiogenesis, while excessive expression leads to an uncontrolled expansion of the vascular bed.

## Part 3: MDM2 Function and the Cardiovascular Cells

Aside from the phenotypic observations in transgenic mouse models, evidence for a cardiovascular role of MDM2 is also supported by studies that have investigated MDM2 and its signaling pathways in endothelial cells, VSMCs and cardiomyocytes.

### Role of the MDM2 Pathway in Primary Endothelial Cells

Endothelial cells form the inner lining of blood vessels. In these cells and MDM2 acts as a negative regulator of TP53, limiting apoptosis (Dai et al., 2012; Brockhaus et al., 2018; Si et al., 2018; Yokoyama et al., 2019). MDM2 could elicit other endothelial functions. Originally, the interest in studying MDM2 in the endothelial cells was initiated by the observation that MDM2 could enhance the production of pro-angiogenic factors in tumor cells. Through its interaction with HIF-1α, MDM2 facilitates angiogenesis by stimulating the expression of VEGF-A in response to hypoxia and growth factors (Skinner et al., 2004; Alam et al., 2009; Park et al., 2011; Patterson et al., 2011). MDM2 might have a similar role in endothelial cells (Song et al., 2012). Since autocrine VEGF-A is crucial to endothelial cell survival (Lee et al., 2007), it is plausible that MDM2 also supports endothelial cell survival through a VEGF-dependent pathway. During angiogenesis, endothelial cells switch rapidly from a quiescent state to a migratory and proliferative state. The observation made by Seccherio and colleagues (2007) that MDM2 antagonist Nutlin-3 inhibits cell cycle progression and cell migration in HUVEC was one of the first lines of evidence that MDM2 could facilitate the activation of endothelial cells to support tumor angiogenesis. Interestingly in this study, the author reported that tumor angiogenesis was inhibited by nutlin-3 independently of TP53. This is an additional piece of evidence that MDM2 angiogenic function might be independent of TP53. We have observed in primary human and mouse endothelial cells that the MDM2 signaling pathway is highly sensitive to pro-angiogenic stimuli. Shear stress, VEGF-A and serum stimulation increased the phosphorylation of MDM2 on Serine 166 (Milkiewicz et al., 2011; Aiken et al., 2016). By expressing the phospho-mimetic form of MDM2 in primary endothelial cells, we showed that Ser166 phosphorylation restrained the expression of FoxO1 target genes known to promote angiostasis, such as Sprouty-2 and Thrombospondin-1 and stimulated endothelial migration (Aiken et al., 2016). In primary endothelial cells, we have found that FoxO1 directly binds to MDM2, an interaction that is increased by shear stress and serum stimulation and correlates with decreased levels of FoxO1 protein (Milkiewicz et al., 2011).

Together these studies support an important role of MDM2 in endothelial cells to maintain survival and to support endothelial responsiveness to pro-angiogenic stimuli.

### Role of the MDM2 Pathway in Vascular Smooth Muscle Cells

#### MDM2-p53 Interaction in Smooth Muscle Cells

The interest in studying the role of MDM2 in vascular smooth cells has emerged from the observation that TP53 inactivation stimulates atherosclerosis (Ihling et al., 1998; Mercer et al., 2005; Mercer and Bennett, 2006). Atherosclerosis is characterized by inflammation and excessive proliferation of VSMCs into the tunica intima. Hashimoto et al. (2011) have clearly demonstrated that disruption of MDM2-TP53 interaction restrained neointimal hyperplasia following vascular injury. Treatment with the small molecule MDM2 antagonist Nutlin-3 suppresses VSMC proliferation and migration. Nutlin-3 promoted cell cycle arrest in G1 in a TP53-dependent manner. *In vivo* Nutlin-3 treatment significantly reduced the intima area and the intimal/media area ratio after vascular injury compared to untreated animals. Nutlin-3 treatment also led to lower levels of inflammation in response to Tumor Necrosis Factor-α (TNF-α). This suggests that inhibition of MDM2 prevented inflammation by restoring the capacity of TP53 to inactivate the NF-κB (Hashimoto et al., 2011).

This pivotal work has been followed by other studies that confirmed that inhibition of MDM2-TP53 interaction can prevent the proliferation of VSMCs. Wu et al. (2014) have demonstrated that the Long intervening non-coding-RNA p21 (LincRNA-p21) and TP53 compete for binding to MDM2 in VSMCs. LincRNA-p21 binding to the RING domain of MDM2 facilitates TP53-p300 interaction, increasing TP53 transcriptional activity in VSMCs. LincRNA-p21 expression appeared to be significantly reduced in atherosclerotic plaque of ApoE^–/–^ mice and in coronary arteries of patients with coronary artery disease. *In vitro*, silencing lincRNA-p21 using siRNAs significantly increased cell viability and proliferation. And *in vivo*, knockdown of lincRNA-p21 in ApoE^–/–^ mice favored the interaction between MDM2 and TP53 and facilitated neointimal hyperplasia. Additionally, Li et al. (2015) have shown that p55γ, a regulatory subunit of PI3K, inhibited the ubiquitination of TP53 by MDM2 in smooth muscle cells. Overexpression of p55γ dramatically reduced the proliferation of VSMCs in culture, increased TP53 protein levels and significantly decreased TP53 ubiquitination. This demonstrates that p55γ activates and stabilizes TP53 by attenuating the capacity of MDM2 to bind to TP53. *In vivo*, delivery of p55γ by adenoviral infection reduced the production of neointima formation post artery injury (Li et al., 2015). Together these studies indicate that enhancement of MDM2-TP53 interaction supports vascular smooth muscle hypertrophy during atherosclerosis.

#### Beyond TP53: HDAC1 as a Downstream Effector of MDM2 in Smooth Muscle Cells

MDM2 is also suggested to play an important role in smooth muscle biology independently of TP53. In fact, MDM2 could mediate vascular calcification through a mechanism involving the ubiquitination of HDAC1 (Kwon et al., 2016). Vascular calcification, the deposition of calcium phosphate, is often associated with the development of atherosclerosis (Giachelli, 2003). Treatment of SMCs with Pi induces calcification. Kwon and colleagues showed that Pi treatment induced the proteasomal degradation of HDAC1, an event that preceded vascular calcification. Pi treatment also upregulated MDM2 and triggered the ubiquitination of HDAC1 by MDM2. Chemical inhibition of MDM2 prevented vascular calcification. The silencing of TP53 did not modulate vascular calcification in this study. This suggests that vascular calcification induced by Pi-treatment requires an MDM2/HDAC1 pathway.

#### Upstream Regulator of MDM2 in Smooth Muscle Cells

Disturbances of the renin-angiotensin-aldosterone system are part of the physiopathology of atherosclerosis, supporting VSMC hypertrophy, proliferation and migration (Nehme and Zibara, 2017). Several pieces of evidence converge to suggest that crosstalk between MDM2 and elements of the renin-angiotensin-aldosterone system could support atherosclerosis by modulating smooth muscle hypertrophy. First, angiotensin II can regulate upstream kinases of MDM2, particularly PI3K and the ERK (Saward and Zahradka, 1997; Li and Malik, 2005; Cooper et al., 2007; Bunni et al., 2011). While the effect of angiotensin II on MDM2 activity in VSMCs has not been directly examined, it is plausible that phosphorylation of MDM2 by Akt or ERK promotes VSMC proliferation. Aldosterone stimulation of MR significantly increased the expression of MDM2 protein in VSMCs (Nakamura et al., 2006; Yan et al., 2018). *In vivo*, chronic treatment of rats with aldosterone increased MDM2 protein level as well as smooth muscle cell proliferation and collagen deposition in the aorta. The silencing of MDM2 significantly reduced smooth muscle cell proliferation. These effects were prevented by eplerenone, an inhibitor of MR. This supports the idea that MDM2 expression is upregulated by aldosterone, then contributing to VSMC hypertrophy (Yan et al., 2018).

The CaM pathway is another strong regulator of smooth muscle hypertrophy, mediating neointimal proliferation after vascular injury (Li and Malik, 2005; Bouallegue et al., 2009; Li et al., 2015). After carotid injury, Akt-dependent phosphorylation of MDM2 on residue serine 166 was significantly reduced in mice null for CaMKII-δ compared to wild-type mice (Li et al., 2011). Less binding between TP53 and MDM2 was observed in VSMC lacking the CaMKII-δ protein. Thus, CaMKII-δ appears to function as an upstream regulator of MDM2 activity in smooth muscle cells. Since CaMKII-δ-KO mice presented with less neointima thickening than wild-type mice, this incriminates CaMKII-δ in provoking VSMC proliferation and neointimal formation, potentially involving activation of the Akt/MDM2 pathway.

### Role of the MDM2 Pathway in Cardiomyocytes

MDM2 plays an important role in maintaining cardiac function by controlling cellular processes and the stress response in cardiomyocytes.

#### MDM2 and the Cardiomyocyte Response to Hypoxia/Reoxygenation

Toth et al. (2006) demonstrated that cardiomyocytes overexpressing MDM2 acquired resistance to hypoxia/reoxygenation-induced apoptosis. Overexpression of MDM2 by adenoviral infection protected primary cardiomyocytes in culture from apoptosis following 12 h of hypoxia and following hypoxia/reoxygenation (12 h of hypoxia followed by 16 h of re-oxygenation). Chemical inhibition of MDM2 by PNC-28, a peptide that mimics the MDM2-binding domain of TP53, resulted in elevated TP53 levels and increased apoptosis under normoxia, hypoxia, and hypoxia/reoxygenation conditions. This suggests that MDM2 promotes cardiomyocyte survival *in vitro* after hypoxia/reoxygenation. Toth et al. (2006) also showed that MDM2 overexpression prevented hypertrophy of primary cardiomyocytes following endothelin-1 or phenylephrine treatment. These *in vitro* results are well aligned with the observations these authors made *in vivo* that the heart of MDM2 hypomorphic mice had cardiomyocytes with increased cross-sectional areas and an increased area of infarction after ischemia/reperfusion (Toth et al., 2006).

#### MDM2 and the Oxidative Stress in Cardiomyoctyes

During oxidative stress, MDM2 might exert both pro-apoptotic and anti-apoptotic functions in cardiomyocytes. Pikkarainen et al. (2009) reported an increased MDM2 expression after oxidative stress. They found that knockdown of MDM2 with an antisense strategy resulted in an increased level of caspase 3, supporting the notion that MDM2 protects against oxidative stress in cardiomyocytes (Pikkarainen et al., 2009). Unexpectedly, Foo et al. (2007) colleagues demonstrated that MDM2 induced ARC protein degradation directly through its role as an E3 ubiquitin ligase. ARC is a 22 kDa anti-apoptotic protein that is found abundantly in cardiomyocytes and skeletal myocytes (Koseki et al., 1998). Overexpression of MDM2 led to a decrease in ARC levels. H_2_O_2_ significantly upregulated MDM2 protein levels and induced significant apoptotic (50–60%) cell death in cardiomyocytes. This was associated with a time-dependent decrease in ARC protein levels. This was due to an MDM2-dependent degradation of ARC. ARC expression was maintained in MDM2/TP53 double knockout cardiomyocytes, while the reintroduction of MDM2 restored ARC degradation, suggesting a TP53-independent function of MDM2. Foo et al. (2007) study supports a pro-apoptotic role for MDM2 in embryonic cardiomyocytes through its regulation of ARC. It is plausible that MDM2 regulates both TP53-dependent and independent pathways to determine the fate of cardiomyocytes after oxidative stress. Further investigations are required to fully elucidate the role of MDM2 in cardiomyocytes during oxidative stress.

#### MDM2 and the Regulation of the β-Adrenergic Signals

MDM2 can regulate the β-AR signal in cardiomyocytes in a TP53-independent manner that involves G Protein-Coupled Receptor Kinase 2 (GRK2) and β-arrestin2 (Li et al., 2009; Jean-Charles et al., 2016, 2017). The β-arrestin and GRK protein families are multi-functional proteins well known for their roles in desensitizing β-AR signaling by blocking G protein coupling, ultimately controlling contractility. In cardiomyocytes, MDM2 can bind and ubiquitinate both GRK2 and β-arrestin2 (Li et al., 2009; Shenoy et al., 2009). While ubiquitination of GRK2 marks it for degradation by the proteasomes, ubiquitination of β-arrestin2 facilitates its binding to signaling kinases rather than promoting its degradation (Shenoy et al., 2007; Jean-Charles et al., 2016). Cardiomyocytes harboring a double knock-down for *Mdm2* and *Trp53* have an impairment of Ca^2+^ handling after stimulation with the β-agonist isoproterenol (Jean-Charles et al., 2017). The polyubiquitination of GRK2 was decreased in the hearts of mice harboring a cardiomyocyte-specific double knockdown of *Mdm2* and *Trp53*, leading to an increase in GRK2 protein levels. These effects were associated with impaired contractility. Since the delivery of the *Mdm2* gene rescued the cardiac contractility, it appears that MDM2 is required for proper signaling of the β-adrenergic receptor in cardiomyocytes (Jean-Charles et al., 2017). Therefore, in cardiomyocytes, proper control of MDM2 interaction with TP53 and other binding partners might be important to fine-tune survival, hypertrophy, and sensitivity to β-adrenergic signals.

### Summary of Knowledge Gained About the Role of MDM2 in Cardiovascular Cell Types

Due to the role of MDM2 as a negative regulator of TP53, it is not surprising that *in vitro* data support a pro-survival role for MDM2 in endothelial cells, VSMCs and cardiomyocytes. However, MDM2 has functions that are independent of TP53 in these cells.

In smooth muscle cells, pro-atherogenic stimuli appear to upregulate MDM2. This upregulation of MDM2 might lead to unwanted proliferation and calcification through the inhibition of TP53 and HDAC1 activities. These actions of MDM2 would ultimately facilitate atherosclerosis. In endothelial cells, MDM2 is crucial to survival and migration, potentially determining the capacity of endothelial cells to initiate angiogenesis. Not only does MDM2 reduce TP53 function to support survival, but it also has a dichotomous action on VEGF-A signals. MDM2 supports VEGF-A expression and is also able to sense this proangiogenic signal to modulate endothelial cell gene expression. Finally, in cardiomyocytes, MDM2 is a crucial regulator of the stress response through mechanisms both dependent and independent of TP53. More importantly, MDM2 also controls hypertrophy and the sensitivity to β-adrenergic signals.

## Conclusive Remarks

Considering the number of small MDM2 inhibitors that are currently under clinical trials, it is crucial to broaden our knowledge of the physiological functions of MDM2 in the cardiovascular system. Many upstream regulators and downstream effectors of MDM2 have cardiovascular functions. The phenotypes of MDM2 transgenic mouse models clearly highlights how important MDM2 is in the cardiovascular system. Studies performed on the three main cell types of the cardiovascular system, i.e., endothelial cells, smooth muscle cells and cardiomyocytes, further support a key role for MDM2. It is highly plausible that MDM2 acts as a central hub for the cardiovascular stress response. How this multitasking E3 ubiquitin ligase operates to coordinate this stress response in the different cells present in the cardiovascular system remains largely unclear. Further investigations are warranted to elucidate how MDM2 are differentially regulated in the cardiovascular system in the context of human health and disease. By delving more into the study of the cardiovascular functions of MDM2, researchers could open new avenues for the treatment and the prevention of cardiovascular diseases, even for the development of cancer treatments where cardiovascular toxicity is curtailed.

## Author Contributions

BL and ER wrote and edited the manuscript. ER proposed the concept of the review to present the most up-to-date evidence regarding the role of MDM2 in the cardiovascular system.

## Conflict of Interest

The authors declare that the research was conducted in the absence of any commercial or financial relationships that could be construed as a potential conflict of interest.

## Abbreviations

α MyHC: α-Myosin Heavy Chain
Akt: Protein Kinase B
ARC: apoptosis repressor with caspase recruitment domain
ATM: ataxia telangiectasia mutated kinase
β-AR: β-adrenergic receptors
CaM: Ca2+/calmodulin
CaMKII- δ: CaM-dependent protein kinase type II delta chain- δ
E2F1: E2 promoter binding Factor 1
ERK: extracellular signal-regulated kinase
ESRR- β / γ: estrogen related receptor β / γ
FoxO: forkhead box O protein
GRK2: G protein-couple receptor kinase 2
GSK3: Glycogen synthase Kinase 3
HDAC1: histone deacetylase 1
HIF1- α: hypoxia-inducible factor 1- α
HUVEC: human umbilical vein endothelial cells
IGF1: Insulin-like growth factor 1
IGFR1: Insulin-like growth factor receptor 1
IRS1: Insulin-receptor substrate 1
KK-Ay mice: crossbred of diabetic KK and lethal yellow mice carrying a heterozygous mutation of the agouti gene
LDLr: low lipoprotein receptor
lincRNA-p21: Long intergenic non-coding RNA-p21
MAPK: Mitogen-activated protein kinases
MDM2: Murine double minute 2
MR: mineralocorticoid receptors (MR)
NF- κ B: Nuclear Factor κ B
NICD1: intracellular domain of Notch1
NOTCH: neurogenic locus notch homolog protein
p55 γ: PI3k regulatory subunit p55 γ
Pdgf β-Cre -ER: tamoxifen-inducible Cre recombinase under the control of the murine platelet derived growth factor receptor β polypeptide promoter/enhancer regions
Pi: inorganic phosphate
PI3K: Phosphotidylinositol 3-Kinase
PGC1- α: Peroxisome proliferator-activated receptor gamma coactivator 1- α
PIGF: placenta growth factor
PPAR- α / γ: peroxisome proliferator-activated receptor α / γ
PTMs: Post-translational modifications
ROS: reactive oxygen species
RSK: Ribosomal S6 Kinase
SM22 α: Smooth Muscle Protein 22- α
Tip60: Histone acetyltransferase KAT5
TP53: Tumor protein p53
VEGF-A: vascular endothelial growth factor A
VSMC: vascular smooth muscle cell.
